# Nondestructive tribochemistry-assisted nanofabrication on GaAs surface

**DOI:** 10.1038/srep09020

**Published:** 2015-03-12

**Authors:** Chenfei Song, Xiaoying Li, Hanshan Dong, Bingjun Yu, Zhiming Wang, Linmao Qian

**Affiliations:** 1 Tribology Research Institute, Key Laboratory of Advanced Technologies of Materials (Ministry of Education), Southwest Jiaotong University, Chengdu 610031, Sichuan Province, P.R. China; 2 School of Metallurgy and Materials, University of Birmingham, Birmingham B15 2TT, UK; 3 Institute of Fundamental and Frontier Sciences, University of Electronic Science and Technology of China, Chengdu 610054, Sichuan Province, P.R. China

## Abstract

A tribochemistry-assisted method has been developed for nondestructive surface nanofabrication on GaAs. Without any applied electric field and post etching, hollow nanostructures can be directly fabricated on GaAs surfaces by sliding a SiO_2_ microsphere under an ultralow contact pressure in humid air. TEM observation on the cross-section of the fabricated area shows that there is no appreciable plastic deformation under a 4 nm groove, confirming that GaAs can be removed without destruction. Further analysis suggests that the fabrication relies on the tribochemistry with the participation of vapor in humid air. It is proposed that the formation and breakage of GaAs-O-Si bonding bridges are responsible for the removal of GaAs material during the sliding process. As a nondestructive and conductivity-independent method, it will open up new opportunities to fabricate defect-free and well-ordered nucleation positions for quantum dots on GaAs surfaces.

Quantum dots (QDs) are three-dimensionally confined semiconductor nanocrystal with quantum effect^1^,^2^. Due to their unique optical and electronic properties, such as photoionization^3^, cavity quantum electrodynamics effect^4^ and quantum size-effect tunability^5^, QDs have attracted extensive research and can be widely used in photodetector^3^, nano-lasers^6^ and 3^rd^ generation solar cells^5^, etc. However, the realization of these applications relies on the ability to manage ordering and positioning of high-quality quantum dot arrays^2^. A popular solution is growing QDs on nanopatterned GaAs substrate by epitaxial processes, where the hollow nanopatterns on GaAs surface serve as nucleation positions^7^. Nevertheless, the defects on nucleation positions can degrade the optical and electrical properties of quantum devices^8^. Therefore, the nondestructive and site-controlled nanopatterning of GaAs surface is a fundamental issue for the quantum technologies.

Many efforts have been devoted to fabricate nucleation positions on GaAs surfaces. For example, photolithography is an efficient method for nanofabrication of GaAs, but the limited resolution, the contaminants induced by wet etching and the complex processes still are tough challenges^2^. Mechanical stamping by indenting diamond tips can directly produce well-ordered nanoholes on GaAs surface^9^. However, since the required Hertzian contact pressure is quite high, plenty of defects may form in the substrate and it is difficult to grow high-quality quantum dots on these defective positions^9^,^10^,^11^,^12^. Recently, anodic oxidation nanolithography based on atomic force microscopy (AFM) has been successfully utilized for nanofabrication on GaAs surface^13^,^14^. This method depends strongly on the conductivity of sample and the endurance of Pt coating on the tip^15^,^16^, so that it is not fit for fabrication on semi-insulating GaAs samples, such as undoped GaAs. In addition, a post-etching process is necessary to remove the oxidation products on the fabrication area, which may cause complex processes and low fabrication efficiency. Therefore, it is essential to develop a nondestructive and straightforward method for fabrication of nucleation sites on GaAs surface.

In the present study, a tribochemistry-assisted nanofabrication method has been developed by directly sliding a SiO_2_ microsphere on GaAs surface in humid air. The capability of this new method is demonstrated by various nanostructures including nanoholes, nanolines, nanoplanes and so on. Cross-sectional transmission electron microscope (XTEM) observation revealed no lattice distortion beneath the fabricated area, which implies that the involved fabrication mechanism should be greatly different from traditional mechanical scratching or cutting. Further analysis suggests that the tribochemistry may have facilitated the removal of GaAs material during the sliding process, resulting in defect-free hollow structures. It is also found that the potential of this one-step nanofabrication method could be realized for GaAs surfaces in different plane orientations and doping types. It is thus expected that this nondestructive tribochemistry-assisted fabrication method will shed new light on the fabrication of high-quality quantum dots on GaAs surfaces.

## Results and discussion

### Tribochemistry-assisted removal of GaAs by SiO_2_ microsphere

It is well-known that grooves could be formed on a surface when the yield of material occurred^17^,^18^. When a diamond tip was used, the lowest limit of the contact pressure for the fabrication of grooves on GaAs surface was about 4.9 GPa, under which the contact area of GaAs yielded (Supplementary Section 1). Since the severe lattice distortion induced by the plastic yield will degrade the properties of the QDs^9^,^10^,^12^, the mechanical cutting is unsuitable for the defect-free nanofabrication on GaAs.

**Figure 1:**
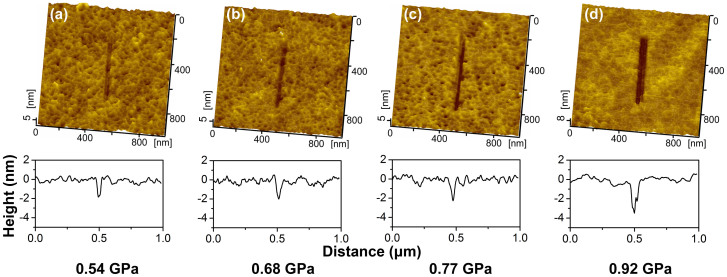
AFM images (top) and cross-sectional profiles (down) of the grooves created on n-type GaAs(100) in air by SiO_2_ tip. The maximum contact pressure *P*_c_ increased from 0.54 GPa to 0.92 GPa, and the scratching cycles *N* was 50.

Previous work suggests that material can be removed by sliding SiO_2_ tip under much lower contact pressure because of the tribochemical wear^19^. It is speculated that the tribochemistry may provide a nondestructive nanofabrication method on GaAs. To verify it, SiO_2_ microspherical probe was selected as fabrication tool to remove GaAs material. As shown in Figure 1, it was found that the effective contact pressure *P*_c_ for the fabrication by a SiO_2_ tip could be as low as 0.54 GPa, which was much less than *P*_y_ = 4.8 GPa for the initial yield of GaAs (Supplementary Section 1). When the contact pressure *P*_c_ increased from 0.54 GPa to 0.92 GPa, the fabrication depth increased from 1.8 nm to 3.3 nm. Compared with the mechanical cutting of sharp diamond tip, the scratching by blunt SiO_2_ tip can make deeper grooves on GaAs surface even under a lower contact pressure. Therefore, the removal mechanism by SiO_2_ tip should be quite different from that by diamond tip. It is thus speculated that the tribochemistry should account for the formation of grooves during sliding SiO_2_ tip^20^,^21^.

### XTEM characterization on the fabrication area

**Figure 2:**
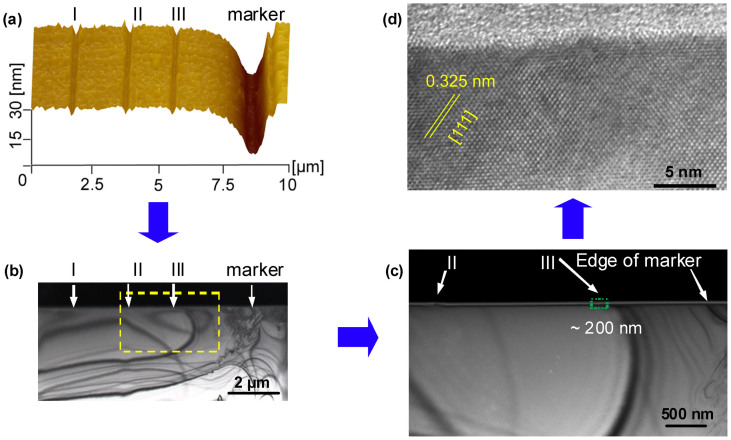
XTEM characterization on the fabrication area. (a) AFM image of the TEM sample, which contains the scratch marker fabricated by diamond tip (denoted as marker) and three nanogrooves fabricated by SiO_2_ tip (denoted as I, II and III); (b) XTEM image of the sample after FIB cutting; (c) Detailed observation of the framed area in (b); (d) HRTEM image of the fabrication area III taken from the green frame in (c).

If the tribochemistry dominates the removal of GaAs by SiO_2_ probe, the fabrication destruction should be avoided since the fabrication is independent of the plastic yield. To demonstrate whether it is a nondestructive method, the lattice of fabrication area was characterized by XTEM. From the AFM image in Figure 2a, the relative position between fabrication area and the marker can be obtained precisely. The mechanical deformation induced by the marker scratch under 5 mN can be easily recognized under TEM such that the fabrication area of I, II and III can be positioned roughly in the TEM images, as shown in Figure 2b. Detailed TEM observation found lightly scratched nanogrooves with a width of about 200 nm at the positions of II and III, as denoted in Figure 2c. A high resolution TEM (HRTEM) lattice fringe image was taken from the fabrication area of groove III, as shown in Figure 2d, to identify if any plastic deformation was induced by 50 cycles scratch under *P*_c_ = 1.01 GPa. Compared to the original surface (Supplementary Figure S2a), although there were few slight distortions on the fabricated surface, no appreciable plastic deformation was observed underneath this 4 nm deep groove. Such distortions can be further avoided when the nanolines are fabricated under a contact pressure *P*_c_ below 0.85 GPa (Supplementary Figure S2b). Therefore, the XTEM results indicate that the defect-free nanofabrication of GaAs can be realized by using the tribochemistry-assisted method with a SiO_2_ probe under a low pressure.

### Mechanism for the nondestructive tribochemistry-assisted nanofabrication

**Figure 3:**
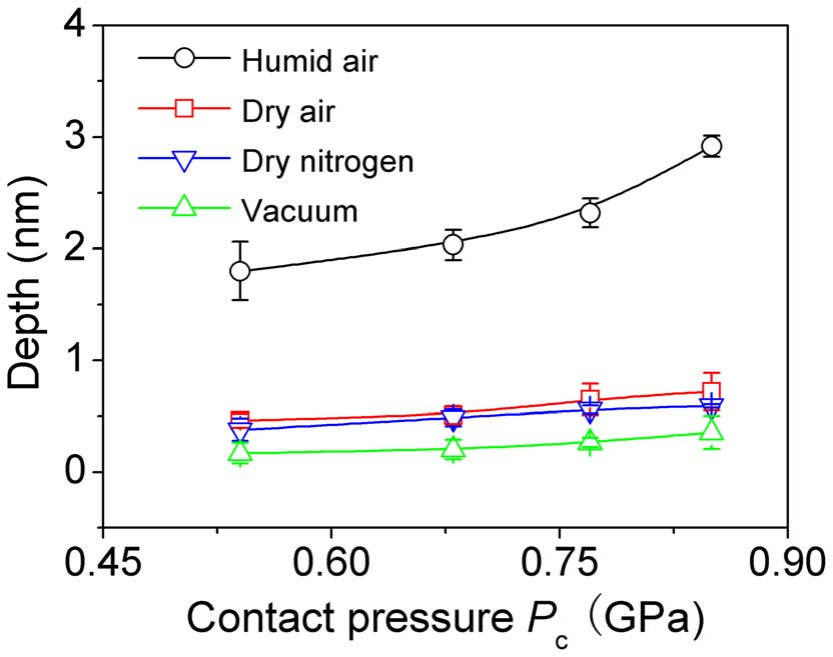
Comparison of the fabrication depth of nanolines on n-type GaAs(100) surface in humid air, dry air, dry nitrogen and vacuum under the same loading conditions. The number of the scratching cycle *N* was 50.

Because the mechanical interaction between a SiO_2_ tip and GaAs substrate is difficult, if not impossible, to produce grooves under a contact pressure far below *P*_y_ = 4.8 GPa, the tribochemistry should be an important mechanism responsible for the removal of materials during sliding. It has been reported that both oxygen and vapor in humid air can affect the tribochemical reaction^19^,^22^. To clarify the respective role of the two gases, the scratching tests were conducted on the same n-type GaAs(100) surface by a SiO_2_ tip under low contact pressures (*P*_c_ = 0.54 ~ 0.85 GPa) in various atmospheres, namely vacuum with a pressure lower than 2.7 × 10^−4^ Pa, dry air (relative humidity *RH* < 1.5%) and dry nitrogen (*RH* < 1.5%). As shown in Figure 3, the fabrication depth increased with the contact pressure under four atmosphere conditions. However, under the same loading condition *P*_c_ = 0.85 GPa, the fabrication depth decreased from 2.9 nm in humid air to 0.7 nm in dry gases, and further decreased to 0.3 nm in vacuum. According to the chemical kinetics, the oxidation reaction between GaAs and O_2_ was related to the striking number per second between the reactants. Assuming that the average fabrication area on GaAs was 10^4^ nm^2^, the striking number of O_2_ on the fabrication area could be estimated as 5.7 × 10^12^ times per second both in humid and dry air because the content of oxygen remained unchanged^23^. Thus, the dramatic reduction of the depth can be mainly attributed to the decrease of the *RH*. However, there were still some shallow nanolines formed in dry air. To further understand the mechanism, dry nitrogen (*RH* < 1.5%) atmosphere was prepared in a renewed vacuum. Although the content of oxygen was down to less than 0.1% in dry N_2_ and the striking number decreased by at least 200 times, the depth of nanolines formed in dry nitrogen was almost the same as that formed in dry air. These results implied that instead of the oxygen, it was the residual vapor that induced the tribochemical reaction and thus produced the nanolines on GaAs surface in dry gases. When the residual vapor was further reduced in vacuum (2.7 × 10^−4^ Pa), the depth of nanoline was 43% of that in dry gases or 10% of that in humid air. Clearly, the vapor in air should have played a key role in the tribochemical reaction during the fabrication process.

**Figure 4:**
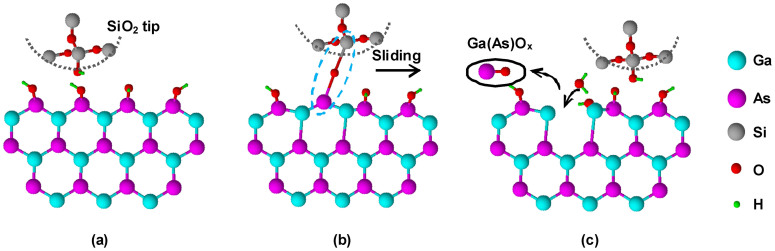
Schematic of the tribochemistry-assisted fabrication on GaAs surface by SiO_2_ tip. (a) Hydroxylation of SiO_2_ and GaAs surfaces in humid air; (b) formation of tip-substrate bonding bridges during sliding; (c) breakage of the bonding bridges and nondestructive removal of GaAs.

Figure 4 schematically depicts the possible tribochemical reaction between a SiO_2_ tip and a GaAs surface during sliding. It is known that water molecules could be adsorbed on the SiO_2_ and GaAs surfaces in experimental humid ambience (*RH* = 50%)^24^,^25^,^26^. When the tip contacted the sample, water meniscus could form on the contact surfaces^27^. With the participation of the vapor and the adsorbed water film, both the SiO_2 _tip and the GaAs surface could be chemically modified by hydroxylation, resulting in hydroxy termination on the contacting surfaces (Figure 4a)^28^,^29^,^30^. Upon sliding contact, the opposite hydroxyls on the contacting surfaces could come in close proximity and collide each others. As shown in Figure 4b, with the help of frictional energy, interfacial bonding bridges between GaAs and SiO_2_ (GaAs-O-Si) might be formed by dehydration reaction^31^,^32^,^33^. During the sliding, GaAs-O-Si bonding bridges would be stretched and the energy was stored in the interfacial bonds. These high energy bonds might readily be broken by adsorbed water through hydrolysis reactions, probably resulting in the formation of oxides such as Ga(As)O_x_ on GaAs and following hydroxylation on other bonds (Figure 4c)^28^,^34^. Such hydrolysis reactions should preferentially occur on GaAs side because the bond strength of Ga-O (353.5 kJ/mol), As-O (481 kJ/mol) or Ga-As (209.6 kJ/mol) was much weaker than that of Si-O (799.6 kJ/mol) (Supplementary Section 3)^35^. As the neighboring Ga-As bonds were hydrolyzed, the oxides products were detached from GaAs substrate, resulting in the wear debris with high oxidation degree (Supplementary Section 4). Finally, such tribo-oxidation products on GaAs can be cleaned easily by ultrasonic water washing^36^. Through this mechanism, the tribochemical reaction and removal of GaAs took place during the continuous tip sliding.

It was reported that only patchy water islands were occasionally observed on mica surfaces at 2% *RH*^37^. Therefore, when the fabrication was conducted in dry gases (*RH* < 1.5%), there should be no reliably water adsorption between the SiO_2_/GaAs contact pair since the GaAs surface was more hydrophobic than mica surface. The slight wear in dry gases might result from the partial water adsorption induced by the residual vapor. While in the vacuum (*RH* = 0), the tribochemical reaction was further restricted due to the absence of the vapor. Since the fabrication depends on the tribochemical reaction during sliding, this method can be named as tribochemistry-assisted nanofabrication.

### Site-controlled nanofabrication on GaAs

**Figure 5:**
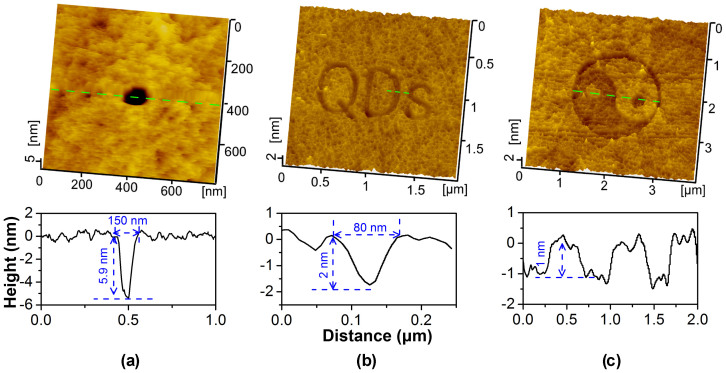
Tribochemistry-assisted nanofabrication on n-type GaAs(100) surface by SiO_2_ tip. (a) Nanohole by scanning-scratch under a contact pressure of 0.92 GPa; (b) Nanowords “QDs” (Quantum Dots) by line-scratch under a contact pressure of 0.68 GPa; (c) Tai Chi pattern by scanning-scratch under a contact pressure of 0.77 GPa.

Finally, site-controlled and defect-free nanofabrication could be realized on a GaAs surface. Based on the controllable scanning of AFM, various types of hollow nanostructures including holes, lines and planes can be designed and produced by this one-step nanofabrication method. As shown in Figure 5a, a nanohole can be fabricated into a GaAs surface by scanning 2 cycles over a 70 nm × 70 nm area under a contact pressure *P*_c_ = 0.92 GPa (*F*_n_ = 2.5 μN). The depth was about 5.9 nm and the diameter was about 150 nm. The letters “QDs” in Figure 5b were written by line-scratch under the conditions of *P*_c_ = 0.68 GPa (*F*_n_ = 1 μN) and *N* = 50, where the average depth of the strokes was about 2.0 nm and the average width was about 80 nm. Tai Chi pattern in Figure 5c was drawn by scanning-scratch at *P*_c_ = 0.77 GPa (*F*_n_ = 1.5 μN) and *N* = 1, where the depth was about 1.0 nm and the diameter was about 2000 nm. Generally, the dimension of the nanostructures on GaAs surface can be controlled by adjusting the fabrication parameters (such as contact pressure and scratching cycles) and the dimension of SiO_2_ microsphere (Supplementary Section 5). When a smaller SiO_2_ microsphere is used, a narrower nanoline is supposed to be fabricated.

Just like the electrochemical reaction during local anode oxidation, tribochemical reaction provides additional energy loading pathway for the material removal^17^. However, the contact pressures *P*_c_ during anodic oxidation by Pt-coated AFM tip was estimated to be 1 ~ 2 GPa when the tip radius was about 50 nm^38^, which was still higher than the contact pressure during the tribochemistry-assisted nanofabrication in this study. This means that the extent of lattice damage induced by tribochemistry-assisted method should not be worse than that by local anodic oxidation. In fact, no appreciable crystal distortion was observed in the tribochemistry-assisted fabrication areas on GaAs surface by a SiO_2_ tip under such low contact pressure in present paper. Moreover, post-etching process can be omitted because hollow structures are formed directly after tribochemistry-assisted nanofabrication. Furthermore, the tribochemistry-assisted method does not rely on the electrical conductivity of sample and AFM probe, thus the fabrication can also be realized on GaAs in different plane orientations and doping types (Supplementary Section 6). The proposed method can provide new chances for the fabrication of defect-free and well-ordered nanostructures on GaAs and other chemical reactive surfaces such as Si^19^,^20^. Moreover, such hollow structures can also be used to define quantum structures underneath GaAs surface through charge depletion^39^.

## Conclusion

Tribochemistry-assisted nanofabrication has been realized on GaAs by sliding a SiO_2_ tip under ultralow contact pressures in humid air. Various hollow nanostructures can be directly fabricated on different types of GaAs surfaces. XTEM results show that the tribochemical reaction during scanning enables us to locally remove the GaAs materials though a nondestructive way. Comparison of the scratching tests in different ambient conditions suggests that the vapor plays a key role in the tribochemical reaction during the fabrication process. As a nondestructive and conductivity-independent method, it will stimulate the new development of the quantum technology.

## Experimental

### Materials

GaAs wafers in different plane orientations and doping types, including n-type GaAs(100), n-type GaAs(111)A, n-type GaAs(111)B and undoped GaAs(100), were purchased from Tianjin Jingming Electronic Materials Co., Ltd., China. By using an atomic force microscopy (AFM, SPI3800N, Seiko, Japan), the root-mean-square roughness of GaAs wafer was measured to be 0.4 nm over a 1 μm × 1 μm area. Before the fabrication, samples were ultrasonically cleaned in methanol, ethanol and deionized water for 10 min. The water contact angle on the samples was measured to be 89° by a contact angle goniometer (DSA100, Krüss, Germany). Then, the samples were placed in AFM chamber with a vacuum capability.

### Fabrication method

**Figure 6:**
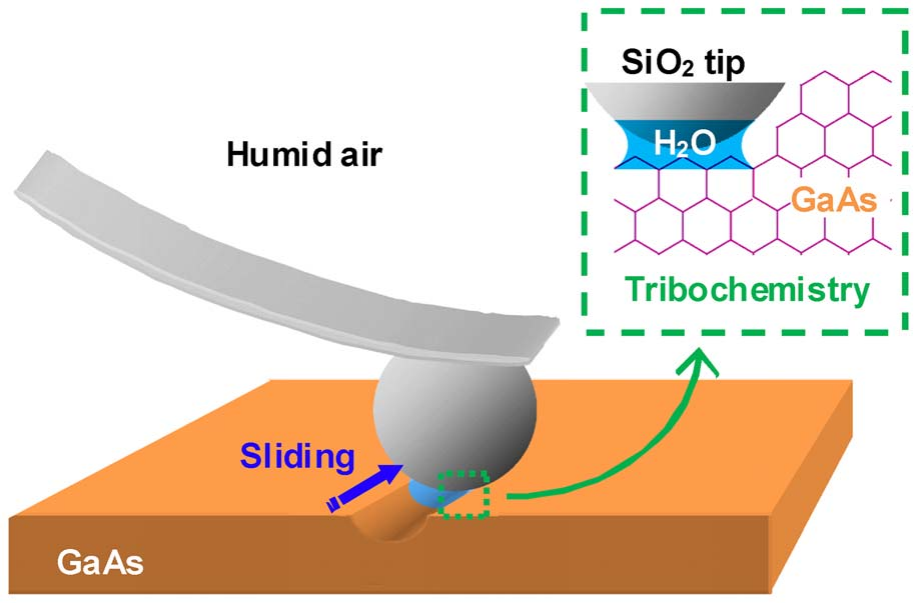
Schematic illustration showing the nondestructive tribochemistry-assisted fabrication on GaAs surface. With the help of tribochemistry in humid air, the nondestructive fabrication can be realized on GaAs surface by sliding a spherical SiO_2_ tip under an ultralow contact pressure.

Spherical SiO_2_ probes (Novascan Technologies, USA) with a radius of 1200 nm were used as fabrication tools. The spring constant of the cantilever was measured as 14 N/m by using a standard cantilever (CLFC-NOBO, Veeco, USA)^40^. Figure 6 schematically shows the nondestructive tribochemistry-assisted nanofabrication process. When a spherical SiO_2_ tip slid on a GaAs surface under a low contact pressure in humid air, a groove could be easily fabricated without destruction. Unlike anodic oxidation nanolithography, the tribochemistry-assisted fabrication process does not need any applied electric field and the surface is not electrically necessary conductive. The upper right inset picture in Figure 6 briefly illustrates the fabrication mechanism involved. Instead of plastic deformation, the water-assisted tribochemistry facilitated the removal of the GaAs. Therefore, the fabricated area could keep its original single crystal lattice. During the fabrication, the sliding speed of the tips was set as 2 μm/s, the temperature was controlled at 20 ± 3°C and the relative humidity *RH* was ranged between 50 ± 5% in humid air, if not specially mentioned. After the fabrication, the topograph of the nanostructure was scanned by Si_3_N_4_ tips with a spring constant of 0.1 N/m (MLCT, Veeco, USA).

### XTEM Characterization

To verify whether the crystal distortion occurs during the tribochemistry-assisted fabrication process on GaAs surface by SiO_2_ tip under a low contact pressure, the cross-sectional transmission electron microscopy (XTEM, JEOL JEM-2100 LaB6, JEOL Ltd., Japan) was used to study the microscopic structure and the potential deformation of fabrication area. The XTEM sample was prepared by using a Quanta 3D FEG focused ion beam miller (FIB, FEI Company, USA). In order to facilitate the FIB cutting across the fabrication area, a mechanical scratch in the depth of about 20 nm was produced by a diamond tip on the GaAs samples as a marker. Beside the marker, a series of nanogrooves in depth of about 4 nm and width of about 200 nm were produced by SiO_2_ tip in humid air.

## Author Contributions

C.F.S. and B.J.Y. realized the fabrication experiments and acquired the original data in this paper. X.Y.L. and H.S.D. did the TEM observation and analysis. Z.M.W. and L.M.Q. have made substantial contributions to the concept and design of this paper. All authors read and approved the manuscript.

## Supplementary Material

Supplementary Information Supplementary Information for Nondestructive tribochemistry-assisted nanofabrication on GaAs surface
