# Supplementary material for: Nondestructive tribochemistry-assisted nanofabrication on GaAs surface
Source: Sci Rep. 2015 Mar 12;5:9020. doi: 10.1038/srep09020 (PMC4356979; doi:10.1038/srep09020)
Supplement: Supplementary Information — for Nondestructive tribochemistry-assisted nanofabrication on GaAs surface [file srep09020-s1.pdf]

Supplementary Information

Nondestructive tribochemistry-assisted nanofabrication on GaAs surface

Chenfei Song,[a] Xiaoying Li,[b] Hanshan Dong,[b] Bingjun Yu,[a] Zhiming Wang*[c] and Linmao Qian*[a]

[a]Tribology Research Institute, Key Laboratory of Advanced Technologies of Materials (Ministry of Education), Southwest Jiaotong University, Chengdu 610031, Sichuan Province, P.R. China

[b]School of Metallurgy and Materials, University of Birmingham, Birmingham B15 2TT, UK

[c]Institute of Fundamental and Frontier Sciences, University of Electronic Science and Technology of China, Chengdu 610054, Sichuan Province, P.R. China

*Corresponding Author: zhmwang@gmail.com, Tel.: +86 28 83202601 and Fax: +86 28 83202601

*Corresponding Author: linmao@swjtu.edu.cn, Tel.: +86 28 87600687 and Fax: +86 28 87603142

1. Fabrication of nanolines on GaAs surface through mechanical cutting by diamond tip

To analyze the role of mechanical interaction in the nanofabrication of GaAs, a diamond tip (Micro Star Technologies, USA) with a curvature radius R of 350 nm was used to fabricate nanolines on GaAs surface. During the fabrication process, the applied normal load F_n was increased from 2 to 16 μN, and the number of line-scratch cycles N was 50. As shown in Figure S1, the surface was wearless under an applied normal load F_n of 2 μN. When the applied normal load exceeded 5 μN, the grooves began to appear and the pile-up of materials was observed at the edges around the grooves. With the further increase of the load, the depth of grooves increased to 1 nm under F_n = 16 μN.

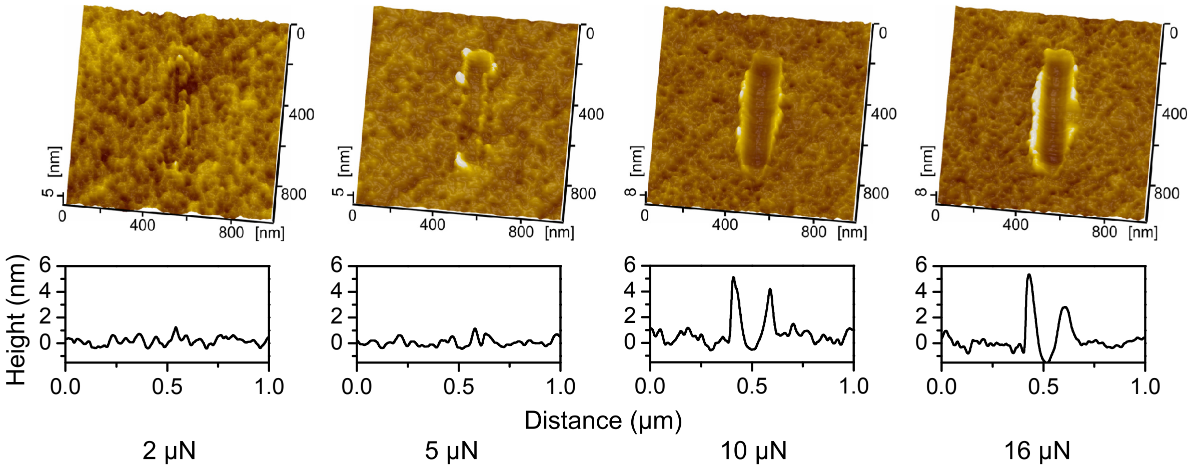

Figure S1. Mechanical cutting on n-type GaAs(100) in air by a diamond tip under various normal loads, the number of scratching cycles *N* was 50.

Since the yield stress of GaAs is 3 GPa,[1] the critical Hertzian contact pressure P_y for the initial yield of GaAs can be estimated as 4.8 GPa.[2,3] As shown in Figure S1, the critical Hertzian contact pressure corresponding to the generation of groove on GaAs can be calculated as 4.9 GPa when F_n = 5 μN, which was close to P_y for the initial yield of GaAs. Therefore, the formation of grooves rests on the yield of GaAs if the mechanical interaction dominates the fabrication process. Similar to mechanical stamping, the mechanical cutting by diamond tip will bring impairment to the GaAs substrate because of the plastic deformation.

However, for the tribochemistry-assisted fabrication by SiO_2 tip in humid air, with the applied normal load F_n= 0.5~2.5 μN, the Hertzian contact pressure P_c can be calculated to be 0.54 to 0.92 GPa, which is much lower than the critical pressure P_y = 4.8 GPa for the initial yield of GaAs. Therefore, only elastic deformation occurred during the fabrication process by SiO_2 tip under the given normal loads. The

analysis indicates that the individual mechanical interaction between SiO_2 tip and GaAs substrate can not produce grooves in this study.

2. Effect of contact pressure on the fabrication defect

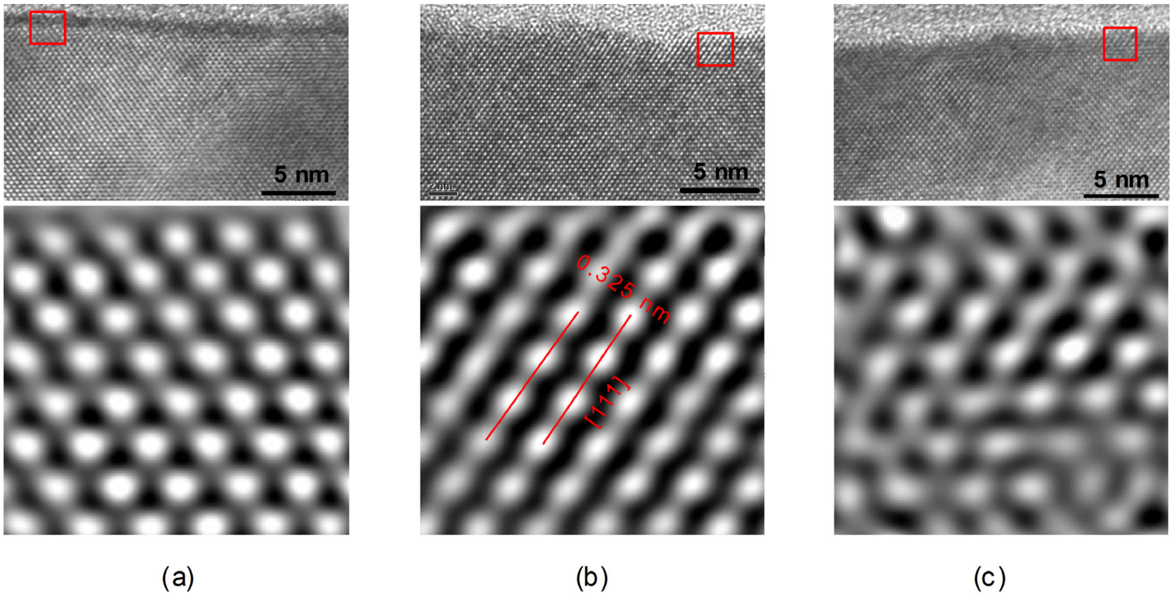

Figure S2. HRTEM observation on GaAs (100). (a) original surface; (b) nanoline fabricated under P_c = 0.85 GPa and N = 100; (c) nanoline fabricated under P_c = 1.01 GPa and N = 50. The higher resolution images were taken from the red frames.

To detect the possible defects in the nanostructures of GaAs surface, the HRTEM observation was performed on the cross section of GaAs samples, as shown in Figure S2. On the original surface in Figure S2a, no defect was observed. In Figure S2b, when the nanoline of 7 nm in depth was fabricated under P_c = 0.85 GPa and N = 100, no defect was detected on the fabrication area. When the contact pressure P_c increased to 1.01 GPa, few defects started to appear in the superficial layer of the 4 nm deep nanoline

(Figure S2c) after scratching 50 cycles. Therefore, the defect-free nanofabrication of GaAs can be realized by using the proposed tribochemistry-assisted method under the contact pressure below 0.85 GPa.

3. Detection of wear on SiO_2 microsphere after fabrication

The SiO_2 tip in this paper consists of fused silica, which has a yield strength of 6.35 GPa.[4] Based on the Tresca yield criterion, the critical contact pressure P_y for the initial yield of SiO_2 can be estimated as 10.2 GPa.[2] During fabrication, since the effective contact pressure (Supplementary Section 1) was much lower than the value of P_y, the SiO_2 should not yield under the selected pressure.

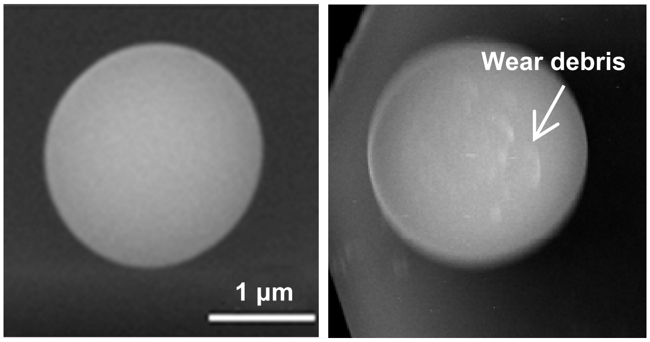

(a) (b)

Figure S3. SEM images of the SiO_2 tip (a) before and (b) after the tribochemistry-assisted nanofabrication on GaAs.

Figure S3 showed the SEM images of the SiO_2 tip before and after the tribochemistry-assisted nanofabrication on GaAs. Before sliding, the tip surface was clean (Figure S3a). After sliding about 5×10^3 cycles, only minor amounts of wear debris were observed (Figure S3b). Since the tribochemical reactions preferentially occurred on GaAs side, these wear debris should mainly come from the GaAs surface.

Therefore, it can be speculated that there was very little surface change of the SiO_2 tip after each experiment under the given conditions.

4. XPS detection on the wear debris of GaAs surface

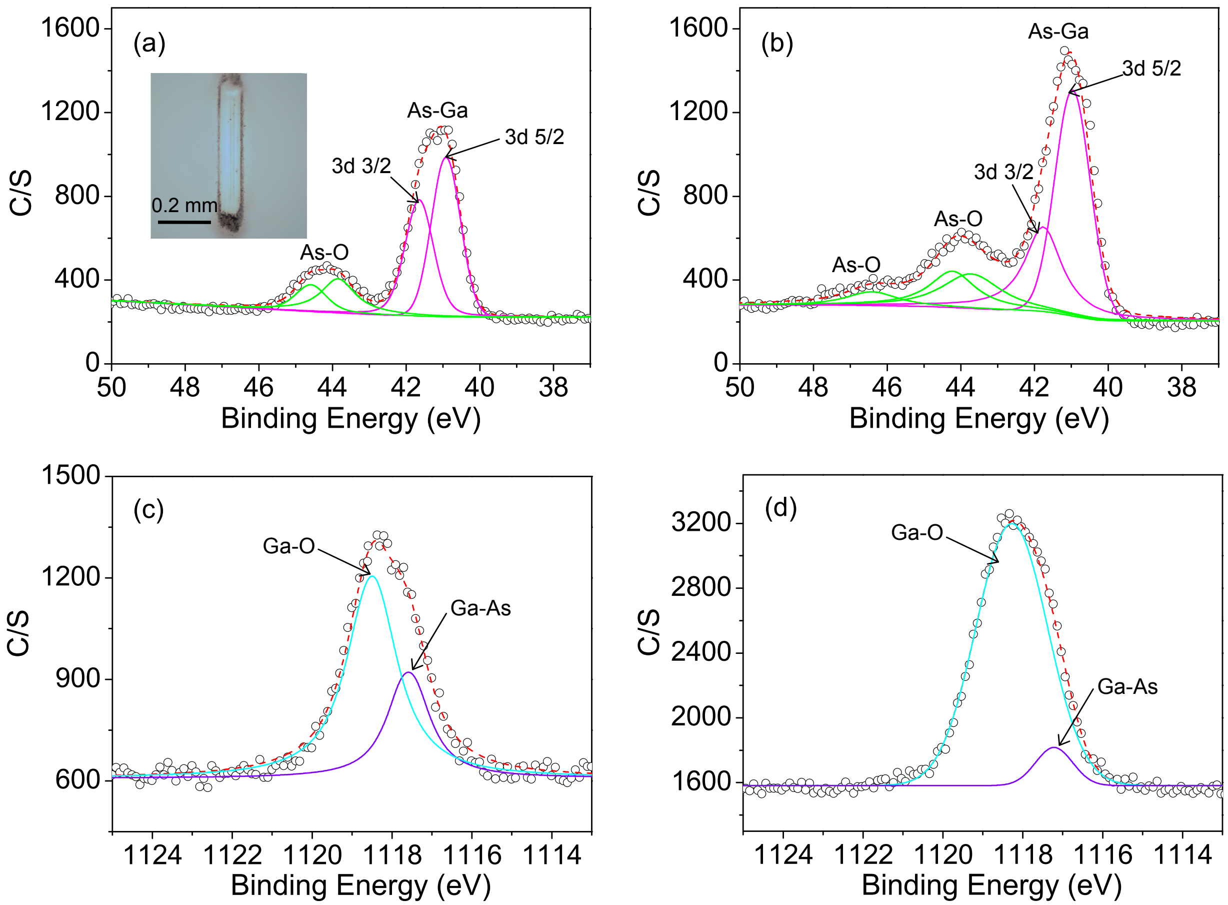

Figure S4. XPS detection on the wear debris and the original surface of GaAs. (a) As 3d spectra on original surface; (b) As 3d spectra on wear debris; (c) Ga 2p spectra on original surface; (d) Ga 2p spectra on wear debris. Binding Energy was calibrated with C1s = 284.8 eV. The inset picture was the optical image of the wear debris surrounding the scratch.

The defect-free lattice in the fabrication area suggested that the GaAs material was not removed by mechanical cutting. It was speculated that the GaAs-O-Si bonding bridges may be broken through hydrolysis reactions, accompanying the formation of oxides such as Ga(As)O_x on GaAs. To verify this speculation, X-ray photoelectron spectroscopy (XPS, PHI Quantera) detection was performed on the wear debris and the original surface of GaAs. The XPS samples were prepared by sliding a SiO_2 ball with radius of 1 mm so that we could collect enough tribochemical products.

As shown in Figure S4a, two peaks were observed on As 3d spectra, the peak centered at about 41.3 eV represented As-Ga and another peak centered at about 44.4 eV represented As-O.[5] The As 3d peaks were assumed with a separation of about 0.7 eV (40.9 eV for 3d 5/2 and 41.6 eV for 3d 3/2).[6] In Figure S4b, an additional peak at 46.5 eV was observed, which might represent higher oxidation states As^{5+}. The ratio of As-O versus total As 3d was 24.5% on the original GaAs (Figure S4a), and it increased to 35.3 % on wear debris (Figure S4b). For the Ga 2p spectra, oxide shoulder was present at a binding energy of about 1118.5 eV (Ga-O). The ratio of Ga-O versus total Ga 2p was 69.8% on the original GaAs (Figure S4c), and it increased to 93.0% (Figure S4d) on wear debris. The increase of the oxides concentration on wear debris suggested that GaAs was removed probably through tribochemical process rather than mechanical cutting.

5. Controlment of the dimension of the nanostructures on GaAs surface

As shown in Figure S5a, when the contact pressure P_c increase from 0.54 GPa to 0.92 GPa, the fabrication depth increase from 1.8 nm to 3.3 nm after liner scanning by 50 cycles. In Figure S5b, as the scratching cycles increased from 10 to 120 under the contact pressure P_c = 0.77 GPa, the fabrication depth

increased from 1.1 nm to 4.5 nm. Therefore, the fabrication depth of nanostructures can be easily controlled by adjusting the contact pressure P_c and the number of scratching cycles N.

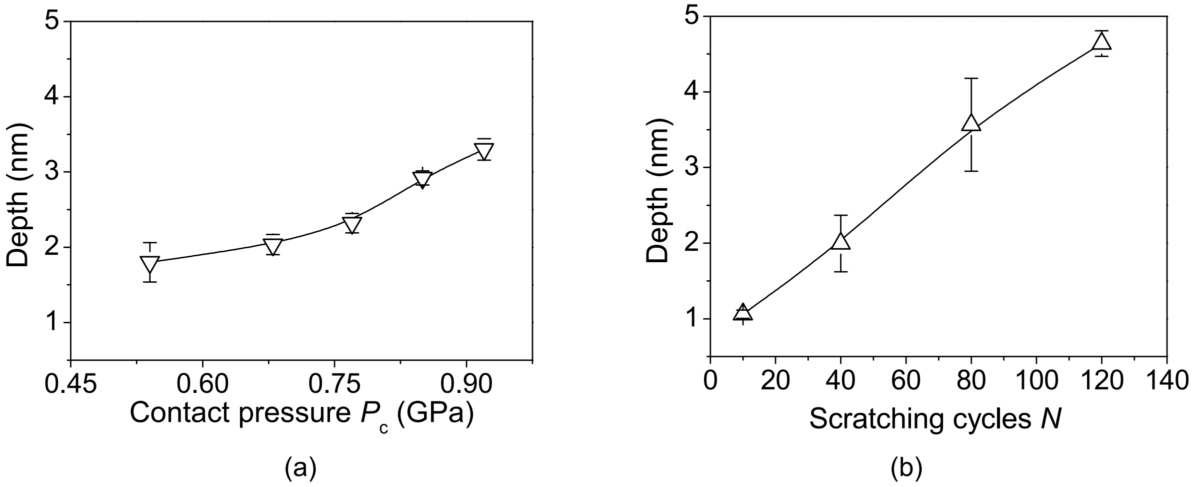

Figure S5. Effect of contact pressure (a) and scratching cycles (b) on the fabrication depths of n-type GaAs(100) in air.

Figure S6 show the variation of the width of nanolines as the function of the contact pressure and the number of scratching cycles. Both the width of nanoline W and the Hertzian contact radius a_0 increase almost linearly with the maximum contact pressure P_c. As a result, there is a linear relationship between W and a_0, namely $W = 2.5a_0$. Therefore, the width of nanoline depends strongly on the contact area of counter pairs. When a smaller SiO_2 microsphere is used, a narrower nanoline is supposed to be fabricated since the Hertzian contact area is smaller.

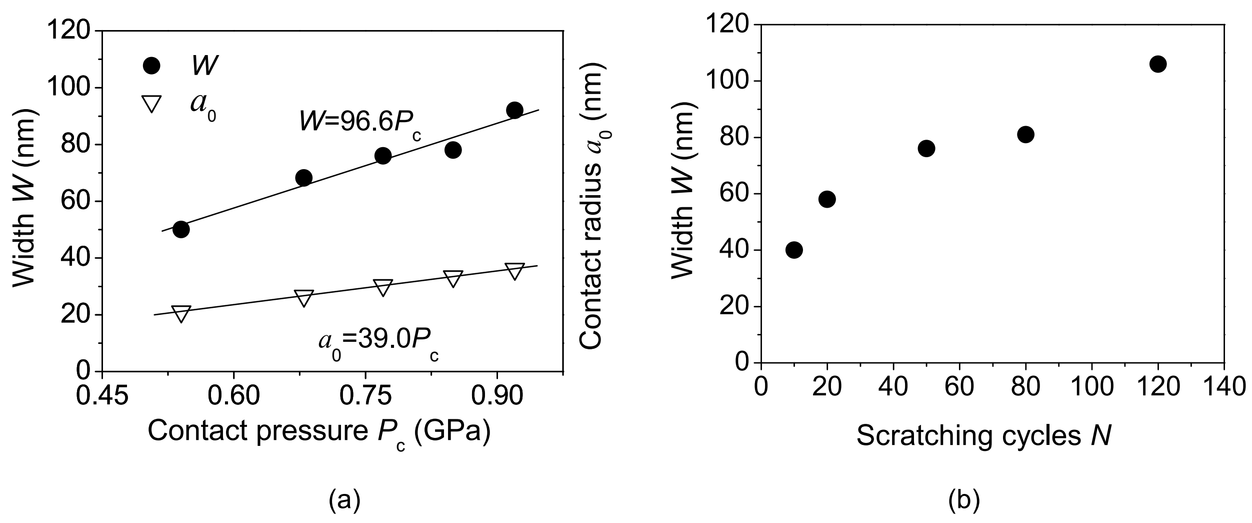

Figure S6. (a) Effect of contact pressure on the width of nanolines on n-type GaAs(100) in air. The number of scratching cycle was 50. (b) Effect of scratching cycles on the width of nanolines on n-type GaAs(100) in air. The contact pressure was 0.77 GPa.

The machining precision of the fabrication may include the minimum linewidth, the precision of width and depth, which were synthetically affected by the size of SiO_2 tip, the precision of AFM scanner and thermal drift of instrument. To fabricate the nanoline with smaller linewidth or higher width precision, a smaller tip should be selected. For the given AFM and probe cantilever, the loading precision was a constant. Therefore, a larger R of SiO_2 tip would result in a smaller change of the contact pressure, and then cause a higher depth precision. Under the given experimental conditions, the minimum linewidth was 30 nm, the width precision was about 5 nm and the depth precision was about 0.2 nm.

6. Nanofabrication on GaAs surfaces in different plane orientations and doping types

To demonstrate the applicability of the tribochemistry-assisted method, the fabrication was practiced on GaAs surfaces in different doping types and plane orientations. As shown in Figure S7, under the same loading condition P_c = 0.77 GPa, the fabrication depth was 4.5 nm on n-type GaAs(100), 5.3 nm on n-type GaAs(111)A, 5.1 nm on n-type GaAs(111)B and 4.8 nm on undoped GaAs(100), respectively. It was noted that the fabrication could be successfully realized on the semi-insulating undoped GaAs(100) surface. Therefore, as a conductivity-independent process, the tribochemistry-assisted method can potentially provide more chances for the fabrication of quantum dots on GaAs.

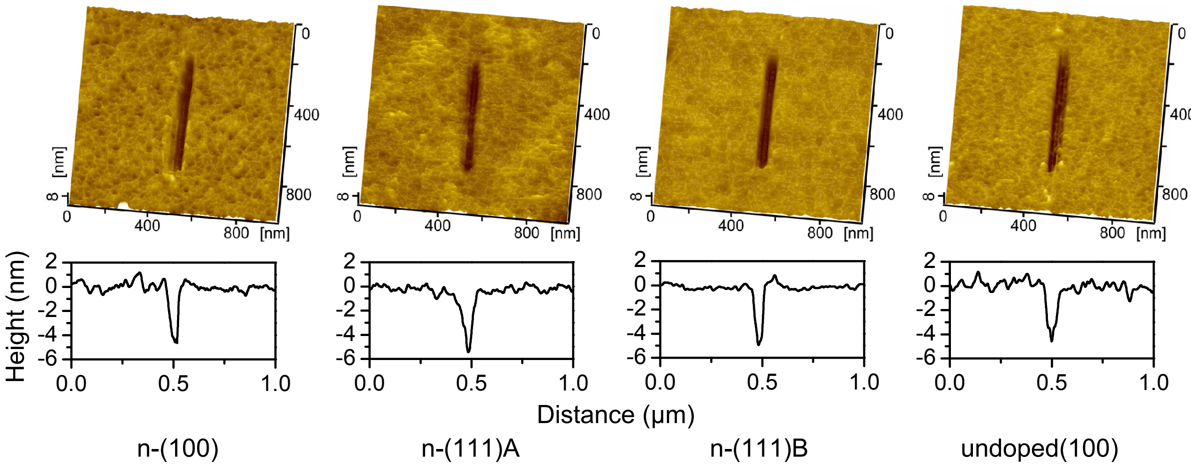

Figure S7. Fabrication of nanolines on n-type GaAs(100), n-type GaAs(111)A, n-type GaAs(111)B and undoped GaAs(100) in humid air by SiO_2 tip. The contact pressure P_c was 0.77 GPa and the number of scratching cycle N was 120.

References

1 Le Bourhis, E. & Patriarche, G. Plasticity of GaAs compliant substructures. *Mater. Sci. Eng. A* **309-310**, 478-482 (2001).

2 Johnson, K. L. *Contact mechanics*. (Cambridge university press, 1987).

3 Grillo, S. E., Ducarroir, M., Nadal, M., Tournie, E. & Faurie, J. P. Nanoindentation of Si, GaP, GaAs and ZnSe single crystals. *J. Phys. D: Appl. Phys.* **36**, L5-L9 (2003).

4 Gadelrab, K. R., Bonilla, F. A. & Chiesa, M. Densification modeling of fused silica under nanoindentation. *J. Non-Cryst. Solids*. **358**, 392-398 (2012).

5 Lee, H. D. *et al.* Reduction of native oxides on GaAs during atomic layer growth of Al_2O_3. *Appl. Phys. Lett.* **94**, 222108 (2009).

6 Hackley, J. C., Demaree, J. D. & Gougousi, T. Interface of atomic layer deposited HfO_2 films on GaAs (100) surfaces. *Appl. Phys. Lett.* **92**, 162902 (2008).
